# Promotion of Erythropoietic Differentiation in Hematopoietic Stem Cells by SOCS3 Knock-Down

**DOI:** 10.1371/journal.pone.0135259

**Published:** 2015-08-07

**Authors:** Yu-xiao Liu, Xing Dong, Feng Gong, Ning Su, Su-bo Li, Hai-tao Zhang, Jia-ling Liu, Jing-hui Xue, Shou-ping Ji, Zhi–wen Zhang

**Affiliations:** 1 First Affiliated Hospital of Chinese PLA General Hospital, Beijing, China; 2 Beijing Institution of Transfusion Medicine, Beijing, China; 3 Third Military Medical University, Chongqing, China; 4 General Hospital of Beijing Military Command, Beijing, China; University of Washington, UNITED STATES

## Abstract

Suppressor of cytokine signaling 3 (SOCS3) plays an important role in mice fetal liver erythropoiesis, but the roles of SOCS3 in human hematopoietic stem cells (HSCs) have not been well investigated. In the present study, lentiviral small interference RNA expression vectors (shRNA) of SOCS3 were constructed and stably transferred into HSCs. We found that SOCS3 knockdown induced erythroid expansion in HSCs. Conversely, Ectopic expression of SOCS3 in progenitor cells blocked erythroid expansion and erythroid colony formation of HSCs. To further explore the involved mechanism, we compared gene expression profiles of SOCS3-shRNA tranduced HSCs with that of control HSCs by whole genome microarrays. The results indicated that cell developmental process related genes, especially hematopoietic lineage-specific genes, associated with the responses to SOCS3 in HSCs.Downexpression of SOCS3 in HSCs or differentiated erythroid progenitor cells induced a transcriptional program enriched for erythroid development relative genes. Our results proved that SOCS3 down-expression induced lineage commitment towards erythroid progenitor cell fate by activation of erythroid-specific gene in HSCs and provided new insight into the mechanism of erythropoietic development.

## Introduction

The availability of red blood cells (RBCs) transfusion is limited by both quantity and the risk of disease [1]. With the repaid development of biology research, generation of RBCs from HSCs and embryonic stem cells (ESCs) may represent an important new resource for blood transfusion [2–6]. Hence, it has great value to establish efficient ways for production RBCs in vitro and study the mechanism in erythropoietic development.

Erythropoiesis is the process by which hematopoietic stem/progenitor cells give rise to lineage-committed erythroid precursors, and terminally differentiate into mature circulating red blood cells. Erythropoiesis is controlled by cytokines in micro-environment and a lot of genes in cells [7–9].

Suppressor of cytokine signaling is a protein family of eight members (SOCS1–7 and CIS) which form a classical negative feedback system to regulate cytokine signal transduction [10].SOCS3 can inhibit the activity of JAK2 kinase and negatively regulate cytokine signaling through the JAK/STAT pathway. Previous studies proved that SOCS3 was involved in placental development, allergic responses, protein ubiquitination and especially in erythropoiesis [11–16]. SOCS3 played a critical role in fetal liver erythropoiesis, SOCS3 deletion resulted in an embryonic lethality with marked erythrocytosisat 12–16 days. In addition, SOCS3 negative regulated the maturation of erythroid cells and inhibited the function of erythropoietin [16–17]. But Roberts et al reported that SOCS3 was dispensable for normal hematopoiesis in the mouse embryo which showed a controversial effect of SOCS3 on erythropoiesis [18]. So, it is still unclear about the role of SOCS3 in the erythroid development of HSCs.

In the present study, we investigated the effect of SOCS3 on erythropoiesis in HSCs by clonogenic progenitor cell assay, flow cytometry, Wright-Giemsa staining and related functional assays. Then we found erythropoietic differentiation of HSCs could be promoted by SOCS3 knockdown and blocked by SOCS3 over-expression. Moreover, we carried a detailed analysis on the underlying mechanism by the HumanHT-12 v4 Expression BeadChip, which have more than 48000 probes. The results revealed that down-expression of SOCS3 increased erythroid-specific gene expression which generated an overview of transcriptional changes in hematopoietic stem cells following SOCS3 knockdown.

## Materials and Methods

### Isolation of human hematopoietic stem cell

Human umbilical cord blood was collected, using a clinically approved method, upon written approval by the mothers. All investigations were approved by the institutional ethics committee of Chinese PLA General Hospital. CD34^+^cells were obtained using magnetic bead separation (Stem Cell Technologies, Cat#18056). More than 95% selected cells were CD34^+^ assessed by FACS.

### In vitro culture of CD34^+^ cells

For expansion, CD34^+^ cells were cultured in Stem Span TM SFEM serum‐free medium (Stem Cell Technologies, Cat#09650) containing 100ng/ml stem cell growth factor (SCF), 50ng/ml thrombopoietin (TPO), 50ng/ml human interleukin-3 (IL-3), and 50ng/ml Fms-like tyrosine kinase3-ligand (Flt3) which all purchased from Peprotech company. In erythroid differentiation experiments, CD34^+^ cells were cultured in SFEM serum free medium supplemented with 5U/ml erythropoietin (EPO), 100ng/ml SCF, 40 ng/mL insulin-like growth factor-1 (IGF-1) and 20ng/ml IL-3 (Peprotech company).

### Plasmid construct

SOCS3 siRNA (siSOCS3-1: 5’-CCAAGAACCTGCGCATCCA-3’; siSOCS3-2: 5’-AGAGCCTATTACATCTACT-3’) were cloned into shRNA vector pSicoR-GFP. The constitutively active human SOCS3 was subcloned from K562 cells and inserted into the pBplv-EGFP vector. Subsequently, lentiviral particles production was performed as described previously [19–20].

### Transfections and Sorting

CD34^+^ cells or erythroid progenitor cells cultured in erythroid differentiation medium for 7day were collected by centrifugation and then resuspended with culture medium containing lentivirus particle and polybrene (a final concentration of 6μg/ml), the cells were plated in 6-well plates at the density of 1×10^6^/ml. Over night, the culture medium was changed and the cells were cultured in normal culture conditions for 72h. 24-72h after transfection, positive cells were sorted by fluorescence-activated cell sorting (FACS), according to the expression of green fluorescent protein (GFP), and then cultured in erythroid differentiation medium.

### Flow cytometric analysis

Cells were incubated with the APC-conjugated and PE-conjugated monoclonal antibodies: anti-human CD34, CD71, CD117, glycophorin A (GPA) and CD45 (B&D Biosciences) for 30 minutes at 4°C, then washed with PBS for 3 times, analyzed by a flow cytometeric analysis using the FACSCalibur (Becton-Dickinson, Mountain View, CA, USA).

### RT-PCR and Real-Time PCR

Total RNA was extracted using TRIzol reagent (Invitrogen) as described by the manufacturer's protocol. RNA was then reverse-transcribed into cDNA by AMV reverse transcriptase (TaKaRa). PCR was performed with rTaq polymerase (TaKaRa). All PCR reactions were performed as follows: 95°C for 5 min; 94°C for 40 s; annealing at various temperatures for 40 s, 72°C for 40 s (25 cycles); 72°C for 10 min, 4°C for 5 min. Real-time PCR analysis was performed on a Bio-Rad iQ5 using the SYBR Green PCR Master Mix (TaKaRa). The following primer pairs were used for the amplication of target mRNAs: *SOCS3* forward primer 5‘ATCC TGGTGACATGCTCCTC’ and reverse primer 5‘CAAATGTTGCTTCCCCCTTA’; *β-Actin* forward primer 5‘ GATCCACATCTGCTGGAAGG’ and reverse primer 5‘ AAGTGTG ACGTTGACATCCG; *EPOR* forward primer 5 ‘TCA AGA CTT A TGGAA CAGGGA TGG’ and reverse primer 5‘GCA GGA TGGATTGGGCAG AC’; *HEMGN* forward primer 5‘AGCTGAA GTGCATGAAA AGGAA’ and reverse primer 5‘ACTTTGGTTA TGCTCCCAGGT’; *CD36* forward primer 5‘GGCTGTGACCGGAACTGTG’ and reverse primer 5‘AGGTCTCCAA CTGGC ATT AGAA’; *IGFBP3* forward primer 5‘AGAGC ACAGATA CCCAGAACT’ and reverse primer 5‘GGTGATTCAGTGTGTCTTCCATT’.

### Western blot analysis

Cells were lysed directly in lysis buffer to collect whole cell extracts. Protein samples were separated on polyacrylamide gels, transferred onto nitrocellulose membrane by iblot (Invitrogen) and detected using horseradish peroxidase-conjugated secondary antibodies and chemiluminesce (SantaCruz) exposure of BioMax film (Kodak). The following antibodies were used: antibodies to SOCS3, actin, STAT5 and p-STAT5 (Tyr 694/Tyr 699) were obtained from Santa Cruz Biotechnology (Santa Cruz, CA, USA). JAK2 and p-JAK2 (Tyr1007/1008) were obtained from Cell Signaling Technology (Beverly, MA, USA).

### Clonogenic progenitor cell assay

Hematopoietic clonogenic assays were performed in 35-mm low adherent plastic dishes using MethoCult GF-H4435 semisolid medium (Stem Cell Technologies, USA) according to manufacturer’s instructions. After 2 weeks, colonies were counted.

### Wright-Giemsa staining

Cells were dropped onto slides and fixed for 20 minutes in 4% paraformaldehyde. Then they were stained with Wright-Giemsa reagents (Fisher Scientific) following manufacturer’s instructions. Cell morphology was analyzed by light microscopy on cytocentrifuged smears stained with Wright-Giemsa on day 21 in erythroid differentiation medium.

### Immuno-fluorescence staining

Cells were fixed in 4% paraformaldehyde for 20 minutes. Then they were washed with PBS, for 3 times, before acquiring Images using the fluorescence microscope DAPI (5μg/ml) was added to dishes.

### Functional Analysis of Hemoglobin

Erythroid cells from shSOCS3-1 CD34 ^+^ cells and normal adult RBCs were collected and were used to characterize the function of hemoglobin. Oxygen equilibrium curves were determined using a hemox-Analyzer, Model B. gas phase gradients were obtained using nitrogen and room air, and the curves were run in both directions

### Whole-genome expression analysis

Total RNA was extracted from hematopoietic cells transfected with shSOCS3 or empty vector (5×10^6^ cells) using Trizol reagent (Invitrogen Life Technologies, Paisley, UK). Genome expression analysis was performed by Illumina Human HT-12 v4 BeadChip (Illumina, San Diego, CA, USA) at the Beijing Qian zhao xing ye Biological Technology Co., Ltd. (Beijing,China).

HumanHT-12 v4 Expression BeadChip targets more than 48000 probes derived from the National Center for Biotechnology Information Reference Sequence (NCBI) RefSeq Release 38 (7 November 2009) and other sources. The beadchips were scanned on the Illumina Bead Array 500GX Reader using the Illumina BeadScan image data acquisition software. Illumina BeadStudio software was used for preliminary data analysis. The preliminary data were normalized using sample averages. To do so, the sample intensities were scaled by a factor equal to the ratio of average intensity of a sample to the average intensity of the given sample. In this article hematopoietic cells transfected with empty vector were regarded as the given sample. Each sample was repeated 3 times.

An Illumina custom algorithm was used to compare shSOCS3-1 CD34 ^+^ cells with control cells. A difference score for a probe (diff score) indicates differential gene expression between these two groups. For each gene, the diff scores of corresponding probes are averaged.

### Statistical analysis

All the data were expressed as means±SD. Statistical significance was determined by using Student’s t-test. Differences were considered significant at P<0.05.

## Results

### The expression of SOCS3 regulated the fate of HSCs

**Fig 1:**
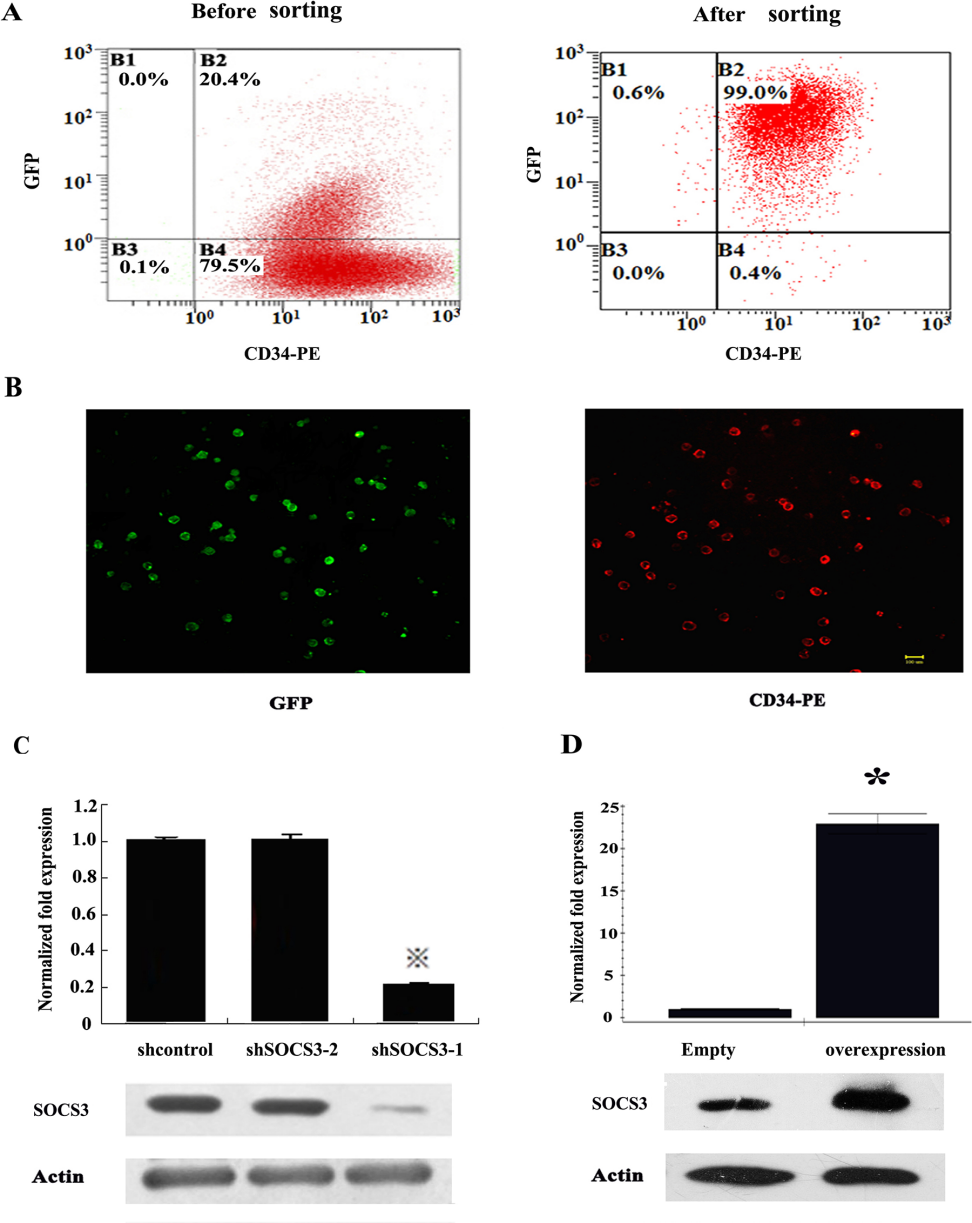
Identification of transfected HSCs. (A) GFP expression in HSCs was analyzed by Flow cytometric. (B) GFP expression in HSCs was analyzed by Immuno-fluorescence staining after sorting. Almost all of CD34+ cells had a strong GFP expression. Bar = 50 μm. Expression of SOCS3 in HSCs was examined by Q-PCR and western-blot after SOCS3 knock-down (C) or over-expression (D). The average of the normalized ratio of the target gene/GAPDH was calculated. The data represent the mean ± SEM from three experiments. ^※^comparing with shControl value<0.05, * comparing with Expression-control value<0.05

To investigate the function of SOCS3 in lineage commitment of HSCs. Lentivirus vectors expressing small interference RNA of SOCS3 were constructed and transferred into HSCs. The vector contained EGFP gene, so lentivirus infection efficiency was estimated by GFP expression. GFP ^+^HSCs were sorted by fluorescence-activated cell sorting (Fig 1A and 1B) After sorting, the expression of SOCS3 in HSCs were examined by real-time PCR and western–blot.Then we found SOCS3 was down-regulated by almost 80% in sorted shSOCS3-1 HSCs at mRNA level. Correspondingly, SOCS3 protein in shSOCS3-1 CD34^+^ cells was also reduced greatly comparing to shcontro1 or shSOCS3-2 CD34^+^ cells (Fig 1C).

**Fig 2:**
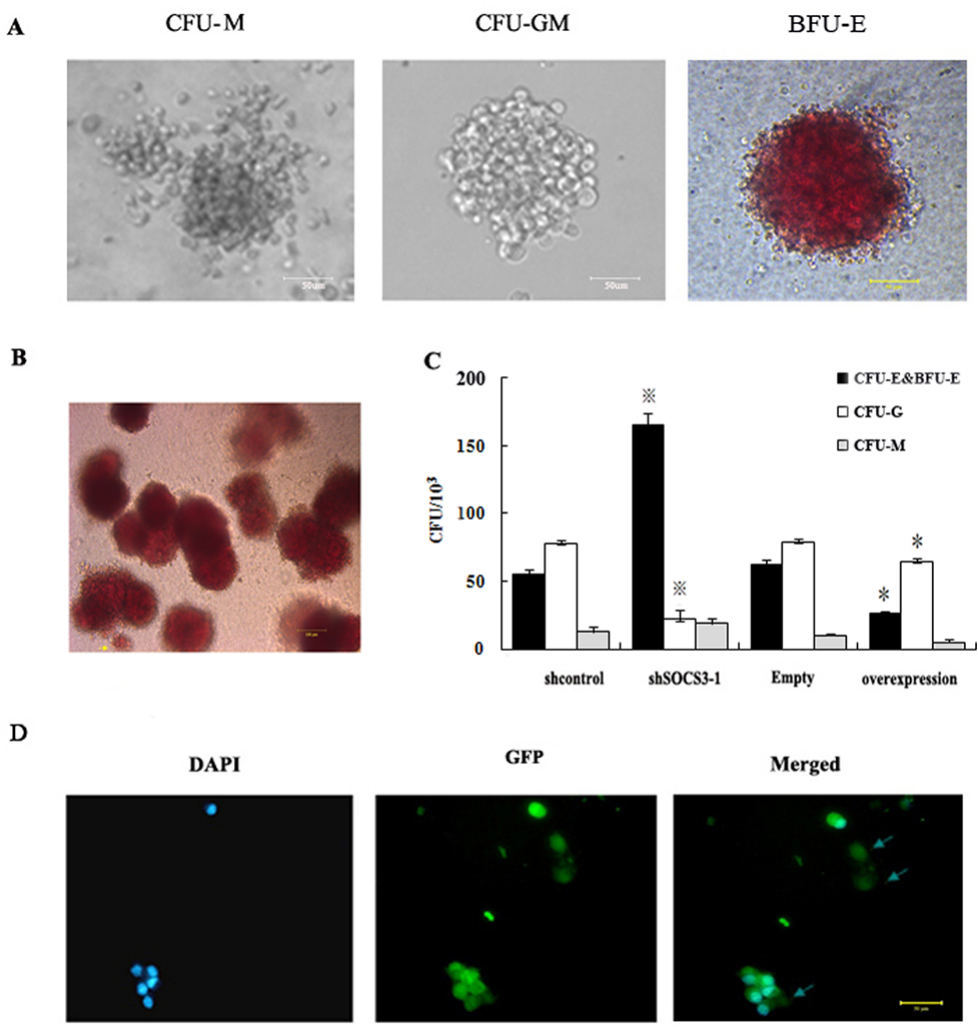
SOCS3 regulated the fate of HSCs. (A) hematopoietic colonies from HSCs. 14 day after seed in methylcellulose, HSCs could form various types of hematopoietic colonies mainly including CFU-M, CFU-GM and BFU-E. Bar = 50 μm. (B) BFU-E and CFU-E derived from shSOCS3-1 CD34^+^ cells. Arrow directed CFU-E. Bar = 100 μm. (C) Quantification of erythroid colonies in different groups. The numbers of erythroid colonies increased by about 3-fold with SOCS3 down-expression and decreased by about 2.5-fold with SOCS3 overexpression. The data represent the mean ± SEM from three experiments.^※^comparing with shControl value<0.05, * comparing with Expression-control value<0.05. (D) Photographs of cytospin preparations of CFU-E from shSOCS3-1 CD34+ cells stained by DAPI. The arrows directed enucleated cells. Bar = 50 μm.

CD34^+^ cells were seeded in methylcellulose. 14 day after seeding, we found shcontro1 CD34^+^ cells could form various types of hematopoietic colonies including colony-forming units–granulocyte-macrophage (CFU-GM), colony-forming units-macrophage (CFU-M) and burst forming unit erythroid (BFU-E) colonies. However, Colony-forming units-erythroid (CFU-E) which represented a later stage of erythroid development could not been seen (Fig 2A). On contrary, the amount of global colony significantly increased in shSOCS3-1CD34^+^ cells, and most of them were erythroid colony (BFU-E colonies accompanying by some CFU-E) (Fig 2B). The numbers of erythroid colony increased by about 3-fold in shSOCS3-1 CD34^+^ cells compared to shcontrol HSCs accompanying by a significant decrease in the number of CFU-GM (Fi.2C). In addition, enucleated red blood cells could be detected in CFU-E from shSOCS3-1 CD34^+^ cells (Fig 2D) which implied that SOCS3 knockdown could promote the global and erythroid colony formation potential of HSCs.

Next, we ectopically expressed SOCS3 in HSCs using lentiviral expression vectors. The expression of SOCS3 in sorted GFP-positive cells increased 20-fold at mRNA level compared to empty vector transduced controls (Fig 1D). When SOCS3 over-expression CD34^+^ cells were plated in semisolid media, the global and erythroid colony formation ability of them was significantly blocked. The numbers of erythroid colonies decreased by about 2.5-fold in SOCS3 over-expression cells compared to empty vector transduced cells (Fig 2C). Most of the colonies from SOCS3 over-expression CD34^+^ cells were CFU-GM which suggested that SOCS3 could control the differentiation of hematopoietic stem/ progenitor cells.

### Knockdown of SOCS3 promoted erythroid determination

**Fig 3:**
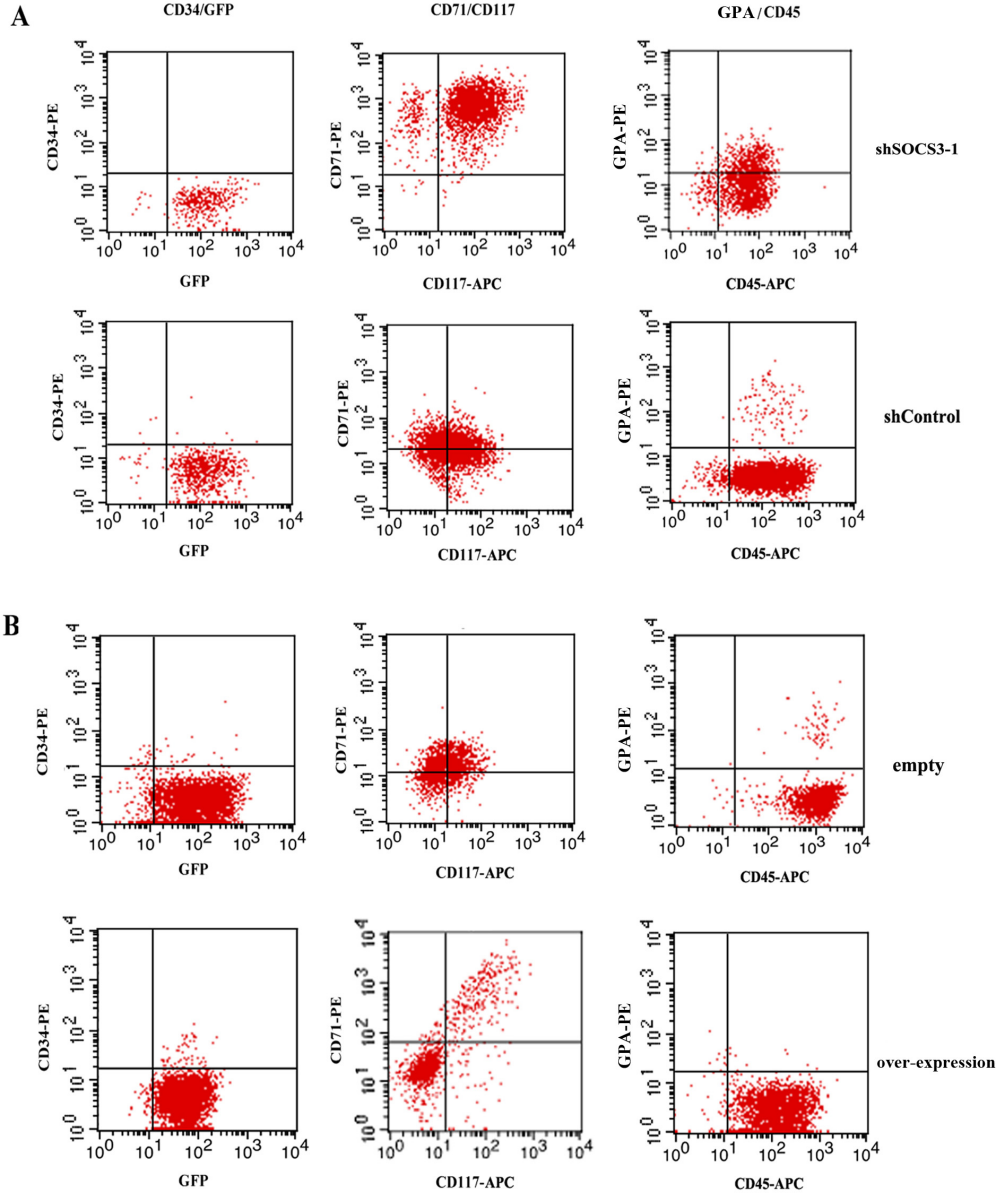
FACS analysis of cell surface markers. On day 7 after liquid culture, cells in different groups were harvested and stained with combinations of fluorescently labeled antibodies against markers characteristic for erythroid lineage.

To further examine the role of SOCS3 in erythroid commitment and differentiation, CD34^+^ cells were cultured in erythroid differentiation medium with a combination of EPO, SCF and IGF-1 which were reported as the necessary factors for proliferation and differentiation of erythroid progenitor from HSCs [21–23]. Differentiation of CD34^+^ cells in different groups was documented by FACS analysis. We found CD34^+^cells rapidly declined and the majority of the cells did not express CD34 on day 7 in all groups (Fig 3 and Table 1). Since CD34 was a marker of early progenitor cells, the results suggested that hematopoietic stem cell started to differentiate as expected. In contrast, the expression of erythroid specific makers increased throughout the rest of differentiation.

**Table 1:** Flow cytometry analysis of cell surface markers on day 7.

Marker	shSOCS3-1	shControl	Over-expression	Empty
CD34 (%)	0.59±0.11	0.64±0.03	0.87±0.16	0.91±0.23
CD71 (%)	95.36±3.31^※^	61.52±3.45	22.11±5.12*	64.36±1.86
CD117 (%)	91.20±7.18^※^	54.02±6.11	21.33±1.04*	56.23±2.59
CD45 (%)	94.15±1.41	92.67±3.12	97.81±2.35	96.12±0.56
GPA (%)	21.23±2.54^※^	2.61±0.82	0.68±0.07*	2.73±0.18

After SOCS3 knock down or over-expression, the cell surface markers expression of cultured erythroid progenitor cells significantly changed. The expression of CD71, CD117, and GPA increased after SOCS3 knock down comparing with shControl, while the CD71, CD117, and GPA decreased after SOCS3 over-expression comparing with Expression-control. The data represent the mean ± SEM from three experiments.

※comparing with shControl value<0.05

* comparing with Expression-control value<0.05

**Fig 4:**
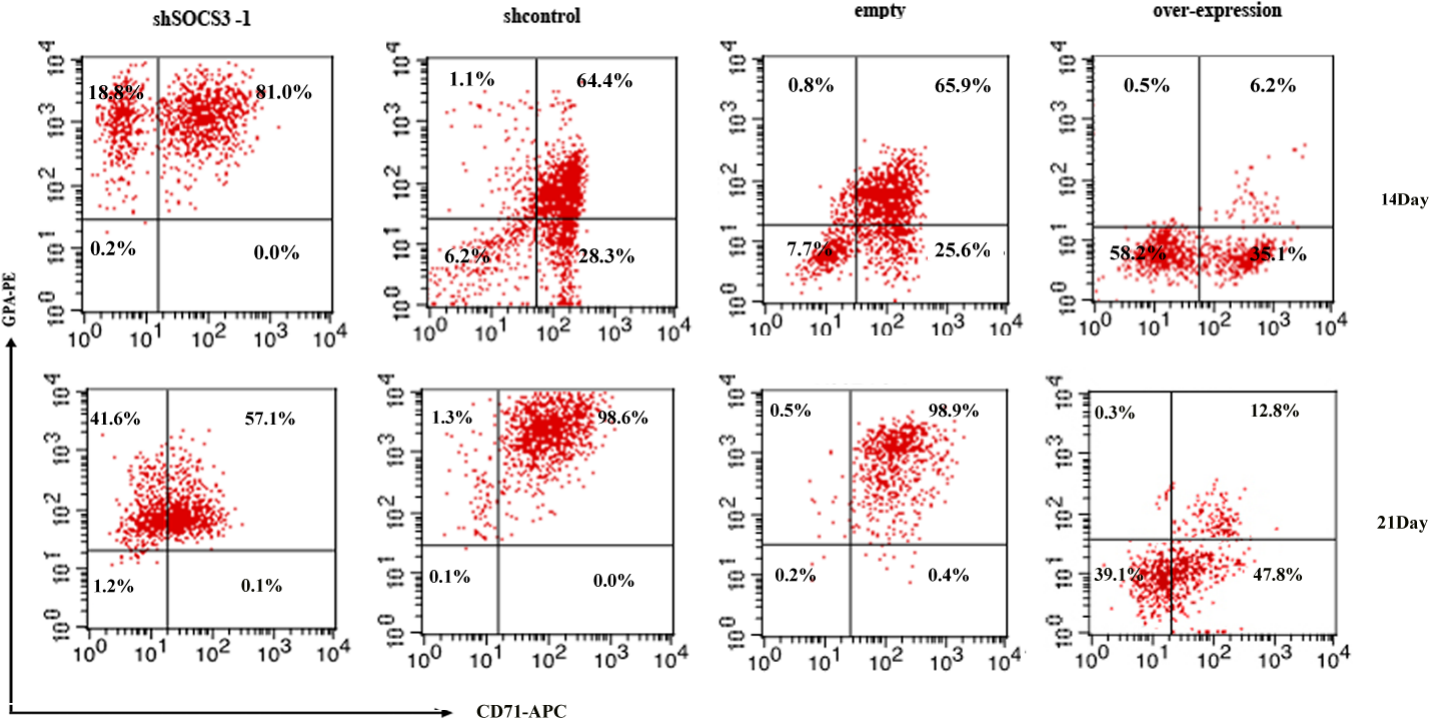
Erythroid specific markers were analyzed by FACS. On day 14 and 21 in liquid culture, cells in different groups were stained with combinations of fluorescently labeled antibodies against markers characteristic for erythroid lineage.

For shSOCS3-1 CD34^+^ cell, we observed a robust erythroid development on day 7 in liquid culture, about 95% cells were CD71 positive, about 91% cells were CD117 positive and about 21% cells were GPA positive. On day 14, the expression of CD71 decreased slightly and the expression of GPA, marker of more mature erythroid cells, increased. On day 21, more than 95% cells were GPA^+^, but the CD71^+^ cells were less than 60% (Fig 4).

For shcontrol vector transduced CD34^+^ cells, CD71 and CD117 positive cells were only about 60%. GPA positive cells were less than 3% on day 7 after sorting (Fig 3A). The CD71 and GPA positive cells increased throughout the rest of erythroid differentiation. On day 21, almost all cells are CD71^+^/ GPA^+^ (Fig 4).

Vice versa, SOCS3 over-expression caused a significant reduction in erythroid cells accompanying by a more than 50% decrease in CD71 and CD117 expression and a about 70% reduction in GPA expression compared to empty transduced CD34^+^ cells on day 7 (Fig 3B). And on day 14, the number of CD71 and GPA positive cells in SOCS3 over-expression group was still much less than that in empty group. On day 21, GPA^+^ cells from SOCS3 over-expression CD34^+^ cells were only about 60% of that from empty transduced CD34^+^ cells (Fig 4). The results suggested that SOCS3 induced skewing of CD34^+^ cells towards an erythroid fate and stable knockdown of endogenous SOCS3 levels promoted erythroid development of hematopoietic stem cells.

### Analysis of erythroid cells from shSOCS3-1 CD34^+^ cells

**Fig 5:**
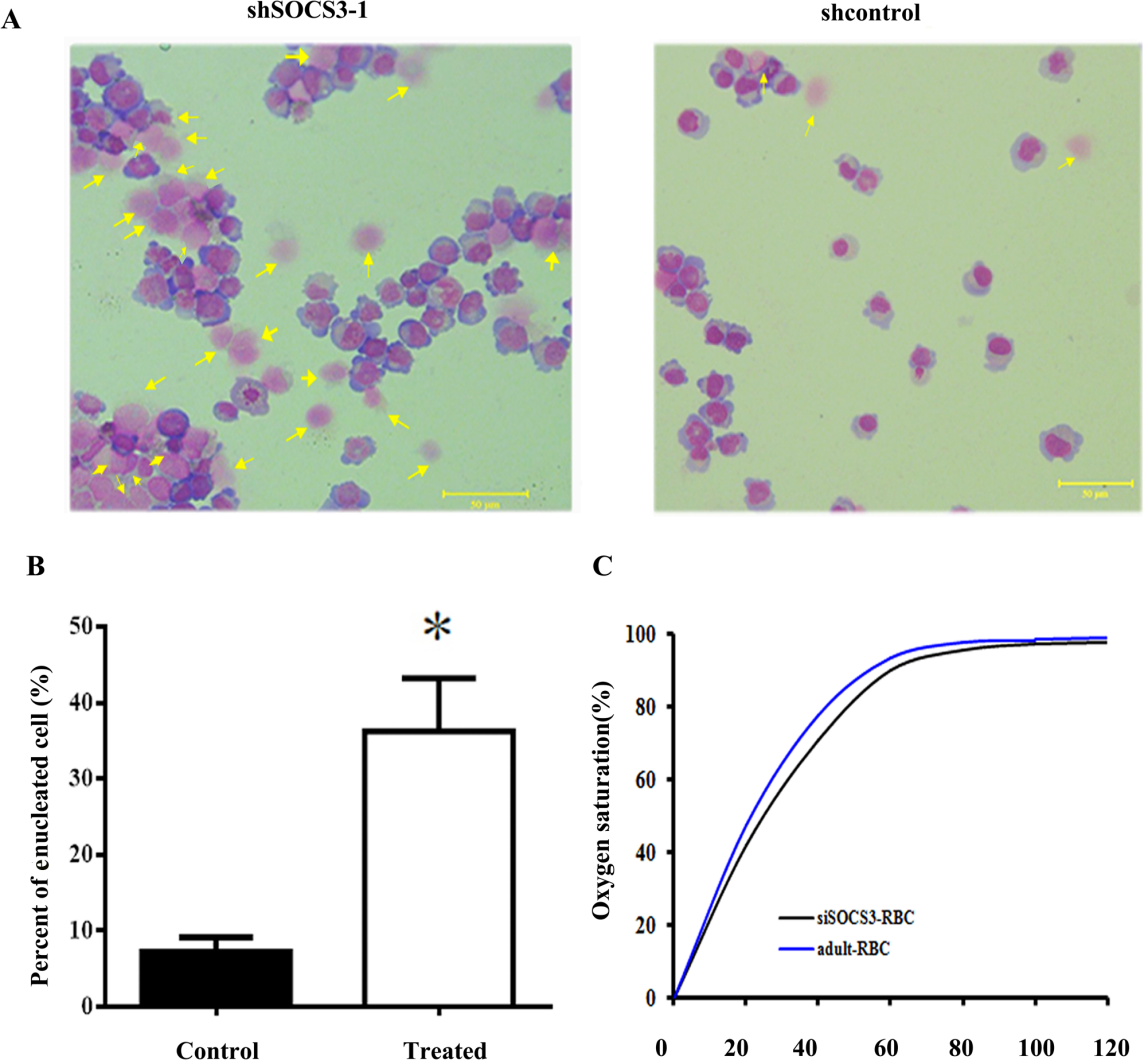
Characterization of erythroid cells from HSCs. (A) Photographs of cytospin preparations of the erythroid cells. Cells from shSOCS3-1 or shcontrol CD34^+^ cells were stained by Wright-Giemsa on day 21 in liquid culture. The arrows directed enucleated cells. Bar = 50um. (B) Statistical analysis for the percent of enucleated cells in different groups. Enucleated cells on day 21 in liquid culture; the percent of enucleated cells was calculated as the ratio enucleated cells / all cells in a field. Data represent mean percent of positive cells ±SEM. n = 5, *p < 0.05. (C) Functional assays of erythroid cells from shSOCS3-1 CD34^+^ cells. Oxygen dissociation curves of RBCs derived from shSOCS3-1 CD34^+^ cells or human adult blood.

To further understanding the characterization of the red blood cells (RBCs) from different groups, we stained them with Wright-Giemsa on day 21 in liquid culture and found that RBCs from shSOCS3-1 CD34^+^ cell had a similar morphology to these from control group. However, more enucleated RBCs could be observed from shSOCS3-1 CD34^+^ cells (Fig 5A). Approximately 40% of erythroid cells from shSOCS3-1 CD34^+^ cells were enucleated. While only 5%–10% erythroid cells from shcontrol CD34^+^ cells were enucleated (Fig 5B). These results confirmed that SOCS3 down-expression promoted erythroid development of HSCs.

Then the oxygen dissociation of shSOCS3-1 CD34^+^ cells–derived erythroid cells were compared with RBCs from normal adult RBCs to assess the function of these cells. As shown in Fig 5C, shSOCS3-1 CD34^+^ cells-derived erythroid cells displayed a similar oxygen dissociation curve to that of normal adult RBCs which implied that the function of RBCs did not be influenced by SOCS3 knock down.

### The effect of SOCS3 down-expression on the gene expression profile of HSCs

**Fig 6:**
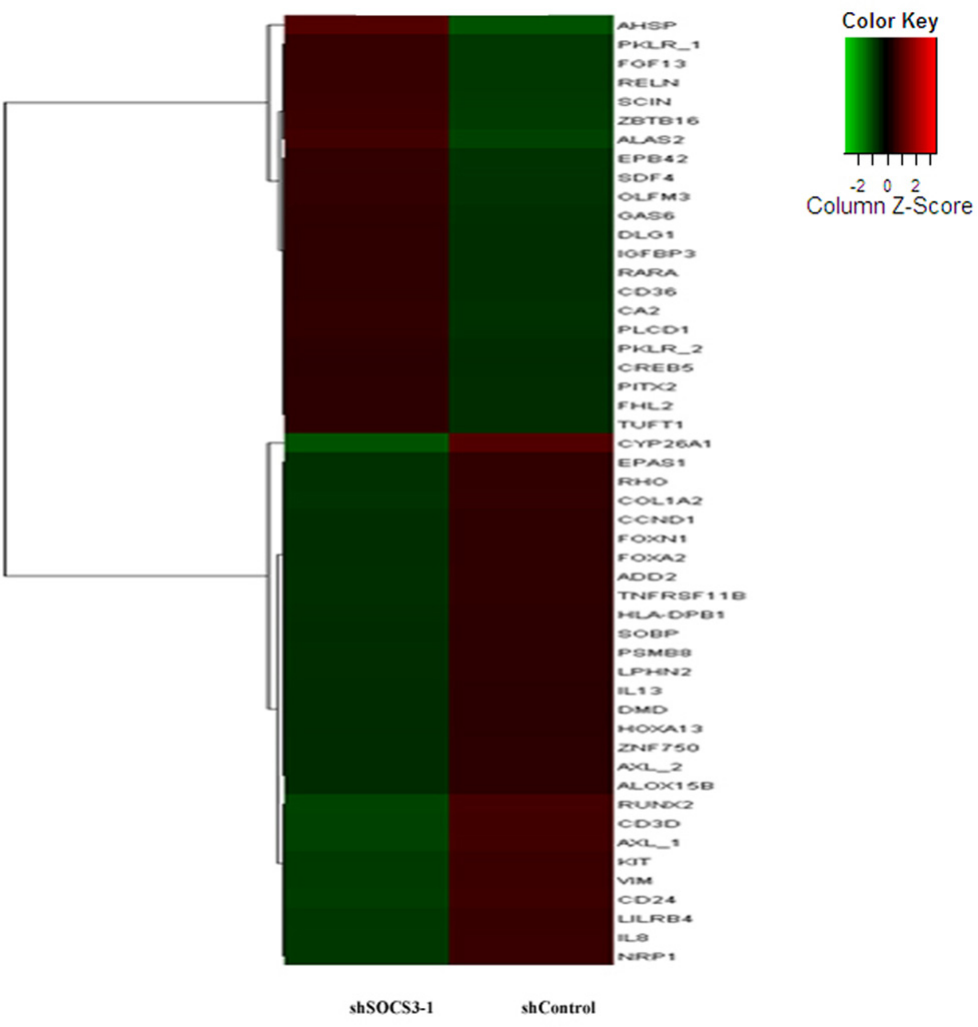
The effect of SOCS3 expression on the gene expression profile of HSCs. Heat-map showing gene expression profiles in CD34+ cells 48 h after transduction with shSOCS3-1 or shcontrol vectors.

To characterize the transcriptional changes caused by SOCS3, the gene expression profile of CD34^+^ cells from human cord blood was determined at 48 h after shSOCS3-1 or shcontrol vector transduction. Total RNA from shSOCS3-1 or shcontrol CD34^+^ cells was hybridized to an Illumina Human HT-12 v4 expression bead array. After normalization, gene expression profile of two groups was compared by using Benjamini Hochberg FDR values. Ultimately, we identified that 313 genes were significant differentially expressed in shSOCS3-1 CD34^+^ cells. Among them, 119 genes were up-regulated and 194 genes were down-regulate. According to cellular function, significantly changed genes were classified (Fig 6, Table 2) as follows: apoptosis (*GAS6*, *AXL*), cell cycle and growth (*CCND1*, *RARA*, *FGF13*, *EPAS1*), cell receptor interaction (*RELN*, *COL1A2*, *INPP1*, *OP52K2*, *OPRM1*), metabolism (*CA1*, *CA2*, *CA8*, *MTTP*), cell differentiation and proliferation (*IGFBP3*, *PBX3*, *SDF4*, *CYP26A1*, *VIM*), immune system responses (*FOXP3*, *ADD2*, *ALAS2*, *CD3D*, *LILRB4*, *EBI2*, *EPB42*) and the erythroid development related genes (*CD36*, *GYPA*, *GYPB*, *HEMGN*.*EPOR*, *EPB42*, *PKLR*).

**Table 2:** The gene expression profile of HSCs after SOCS3 knockdown.

Up regulated gene		Down regulated gene	
Gene name	logFC	Gene name	logFC
*CA1*	2.15	*NTS*	-2.17
*AHSP*	1.95	*ANKRD19*	-1.9
*GYPE*	1.81	*CYP26A1*	-1.94
*GYPB*	1.67	*SLC44A1*	-1.83
*GYPA*	1.61	*LOC63201*	-1.73
*RUNNX2*	1.59	*INPP1*	-1.56
*ALAS2*	1.54	*AXL*	-1.51
*EFCBP1*	1.57	*FAM22E*	-1.54
*CD36*	1.57	*FAM113B*	-1.4
*HEPACAM2*	1.55	*MTTP*	-1.49
*SMAD5*	1.39	*LOC64482*	-1.42
*ZBTB16*	1.38	*LOC63201*	-1.73
*FGF13*	1.29	*INPP1*	-1.56
*PKLR*	1.28	*AXL*	-1.51
*TUBB1*	1.27	*CD3D*	-1.52
*LOC100131895*	1.26	*CD24*	-1.49
*OPRM1*	1.26	*PSMB8*	-1.48
*EPB42*	1.25	*EBI2*	-1.48
*CA8*	1.24	*FNDC8*	-1.36
*OP52K2*	1.23	*VIM*	-1.35
*GAS6*	1.19	*APOBEC4*	-1.35
*SDF4*	1.17	*HPVC1*	-1.35
*CA2*	1.17	*PBX3*	-1.34
*EFNA3*	1.16	*SORL1*	-1.32
*HEMGM*	1.13	*ADKD55*	-1.32
*PLCD1*	1.12	*IL8*	-1.31
*RARA*	1.11	*TMEN140*	-1.29
*CEP63*	1.11	*CCND1*	-1.28
*IGFBP3*	1.10	*COL1A2*	-1.176
*EPOR*	1.09	*ADD2*	-1.19

After SOCS3 downexpression, top 60 down- or up-regulated transcripts in CD34+ cells were shown in table2. Fold changes from three independent experiments relative to shcontrol are indicated.

**Fig 7:**
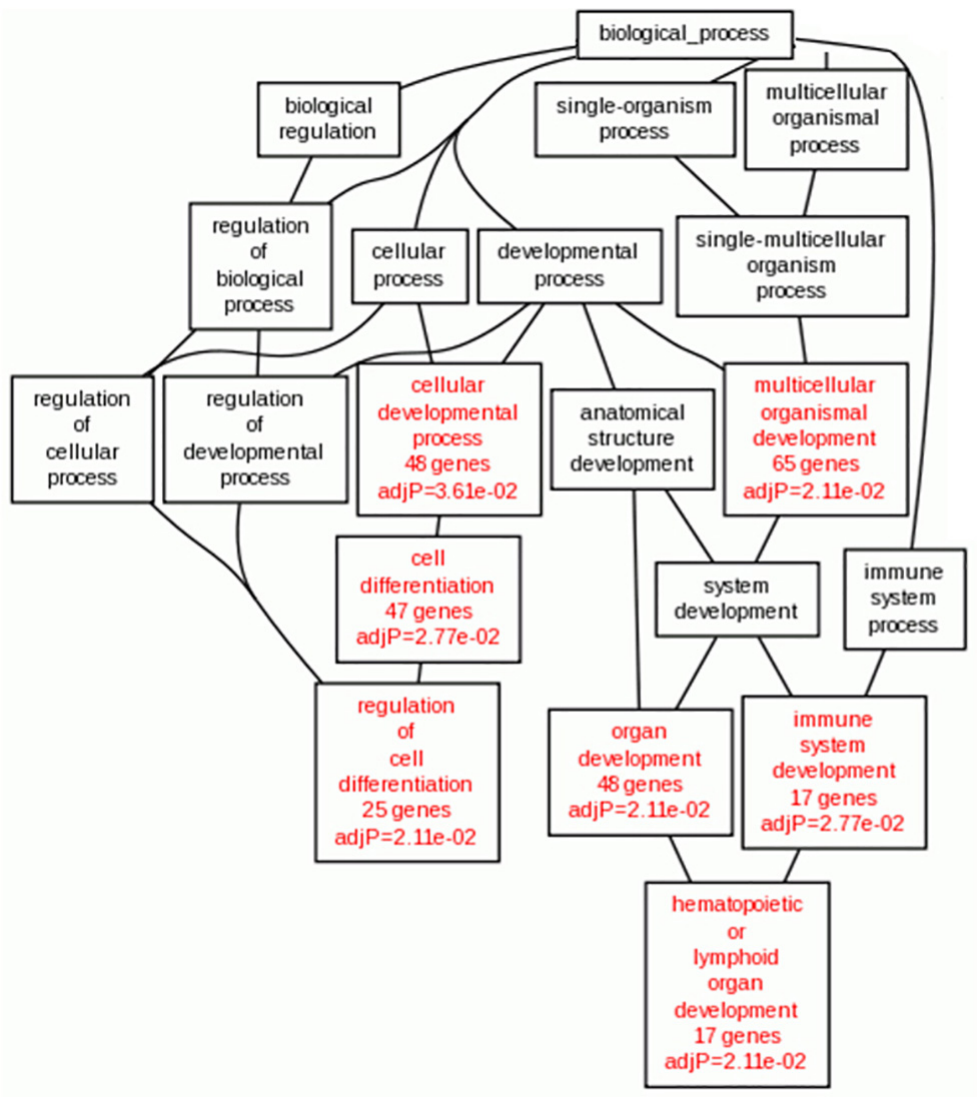
GO analysis of SOCS3-induced gene expression alteration in HSCs. Branches of the GO hierarchical tree with significantly enriched GO terms are indicated in red boxes, Insignificant GO terms within the hierarchical tree are shown as white boxes.

Next, we investigated SOCS3-induced gene expression alteration in HSCs through GO analysis (Fig 7). The results indicated that organ and cell development signaling pathway were mainly associated with responses to knock down of SOCS3 in HSCs.

### Identification of the effect of SOCS3 knockdown on erythroid transcriptional program

Then lentivirus vectors expressing small interference RNA of SOCS3 were also transferred into differentiated erythroblasts on day 7 in liquid culture. We selected four possible candidate genes including *CD36*, *HEMGN*, *EPOR*, *IGFBP* and examined the expression of these genes in HSCs and differentiated erythroblasts after SOCS3 knockdown. *CD36* was the marker for erythroid progenitor [24]. *HEMGN* (also called *EDAG*) and *EPOR* could positively regulate erythroid differentiation [25–26]. *IGFBP-3* could promote the proliferation of primitive CD34^+^ hematopoietic cells. Then we found that SOCS3 knockdown could promote the expression of erythroid differentiation and proliferation related genes not only in HSCs but also in differentiated erythroblasts (S2 Fig). Taken together, these findings further confirmed that SOCS3 down-expression induced a progenitor fate in HSCs with a bias towards the erythroid lineage.

## Discussion

Erythropoiesis at molecular level is driven by a combination of transacting factors that act in concert to direct the genes expression for erythroid-specific proteins [27]. The critical roles of SOCS3 in fetal erythropoiesis and erythroid progenitors maturation have been demonstrated [14–16], while little is known about the role of SOCS3 in erythroid development of HSCs.

In previous study, we proved that K562 cells could be induced into erythroid lineage cells more easily after silencing of SOCS3 [28]. Here, we showed that SOCS3 was important for the lineage commitment of HSCs towards the erythroid. And it was a feasible method to product large numbers of erythroid cells from cord blood-derived HSCs in vitro by SOCS3 knock down.

First, we found erythroid colony formation in HSCs from human cord blood correlated with SOCS3 expression. SOCS3 knockdown resulted in significant increase in the number and the percent of erythroid colonies and SOCS3 gene over-expression almost completely abrogated erythroid colony formation of HSCs.

Then, we induced HSCs into erythroid cells in the serum-free medium with EPO, SCF and IGF-1 after SOCS3 knock down or over-expression. 7days after induction, almost all the cells from shSOCS3-1 CD34^+^ cells were CD71 and CD117 positive which were highly expressed on early erythroid cells. And more than 20% cells from shSOCS3-1 CD34^+^ expressed matured erythroid specific cell surface marker-GPA. However, for SOCS3 overexpression group, CD71 or CD117 positive cells were less than 60% and few GPA positive cells could be found after 7 days induction. On 21day in liquid culture, we found almost all erythroid cells from shSOCS3-1 CD34^+^ cell expressed GPA. In addition, many GPA^+^ cells became CD71^−^ cells. To our knowledge, it was as a sign of cell cycle arrest and terminal maturation to erythrocytes [29]. On contrary, GPA^+^ cells from SOCS3 over-expression CD34^+^ cells were less than 14% at same culture condition.

Erythroid differentiation and maturation were also documented by Wright-Giemsa based the morphological analysis. We found that SOCS3 knock down promoted enucleation of HSCs. As shown in Fig 5, more enucleated RBCs from shSOCS3-1 CD34^+^ cells were detected at day21 in liquid medium compared with control cells which indicted erythropoietic differentiation of HSCs could be promoted by SOCS3 knock-down. A critical issue for clinical application of stem cells-derived RBCs is whether they possess the normal capability of carrying oxygen. We demonstrated here that erythroid cells derived from shSOCS3-1 cord blood HSCs possessed comparable oxygen equilibrium curves to that of normal adult RBCs. These results implied that the shSOCS3-1 HSCs-derived RBCs have same oxygen carrying function as normal adult erythrocytes.

Furthermore, we investigated the roles of SOCS3 in the erythroid fate by analysis the expression profiling in SOCS3 down-expressing CD34^+^cells by gene expression bead array. The result indicated that SOCS3 knock-down increased the expression of multiple erythroid-specific genes and down- regulated genes controlling lymphoid differentiation. To validate the precision of gene expression bead array, real-time RT-PCR was performed on four erythroid development relative genes. The results showed that SOCS3 knockdown could accelerate normal erythroid transcriptional program by promoting the expression of erythroid related genes both in HSCs and in differentiated erythroblasts. In addition, Although there were small differences in the fold change values between two methods of measurement, the data from real-time PCR were in agreement with that from bead array (S2 Fig, Table 2) which strongly supporting the reliability of gene expression bead array analysis.

At last, we examined the effects of SOCS3 over/down-expression on JAK-STAT signaling in HSCs. As illustrated in S1 Fig, stronger expression of p-JAK2 and p-STAT5 were detected in shSOCS3-1 HSCs. On contrary, the level of p-JAK2 and p-STAT5 was inhibited by SOCS3 over-expression, These results were in accordance with the previous studies [12,29].

In conclusion, we show that in hematopoietic progenitor cells, SOCS3 is critical for the commitment towards the erythroid lineages by inducing expression of lineage-specific genes. Expression profiling of CD34^+^ cells after SOCS3 down regulation revealed transcriptional change in genes with mainly related with erythroid cell lineage commitment or differentiation. Down-expression of SOCS3 in hematopoietic stem cells could promote erythroid differentiation of HSCs. In previous studies, stromal cell lines or a variety of hematopoietic growth factors and cytokines were indispensable for in vitro production of erythrocytes from HSCs [30–33].

Here we proved it was a feasible procedure to yield erythroid progenitors from HSCs rapidly and efficiently by altering the expression of specific gene. Our study may be valuable for the futher study of erythropoietic development. However, the accurate molecular mechanism of this inducing system is still unclear. In addition, more safe method to knock down SOCS3 in hematopoietic stem cells is needed. These will be the certain focus of future research.

## Supporting Information

S1 Fig The effect of SOCS3 on JAK/STAT pathway. The effect of SOCS3 on JAK/STAT pathway in HSCs was examined by western-blot after SOCS3 knock-down or over-expression.
(TIF)

S2 Fig The effect of SOCS3 knockdown on erythroid associated gene. Real-time PCR analysis for the expression of *CD36*, *HEMGN*, *EPOR*, *IGFBP* in HSCs (A) or in erythroblasts on day 7 (B) in liquid culture after SOCS3 knockdown. The average of the normalized ratio of the target gene/GAPDH was calculated. The data represent the mean ± SEM from three experiments. * P<0.05 as compared with shcontrol groups.
(TIF)
