# Supplementary figures and images for: Promotion of Erythropoietic Differentiation in Hematopoietic Stem Cells by SOCS3 Knock-Down
Source: PLoS One. 2015 Aug 7;10(8):e0135259. doi: 10.1371/journal.pone.0135259 (PMC4529111; doi:10.1371/journal.pone.0135259)

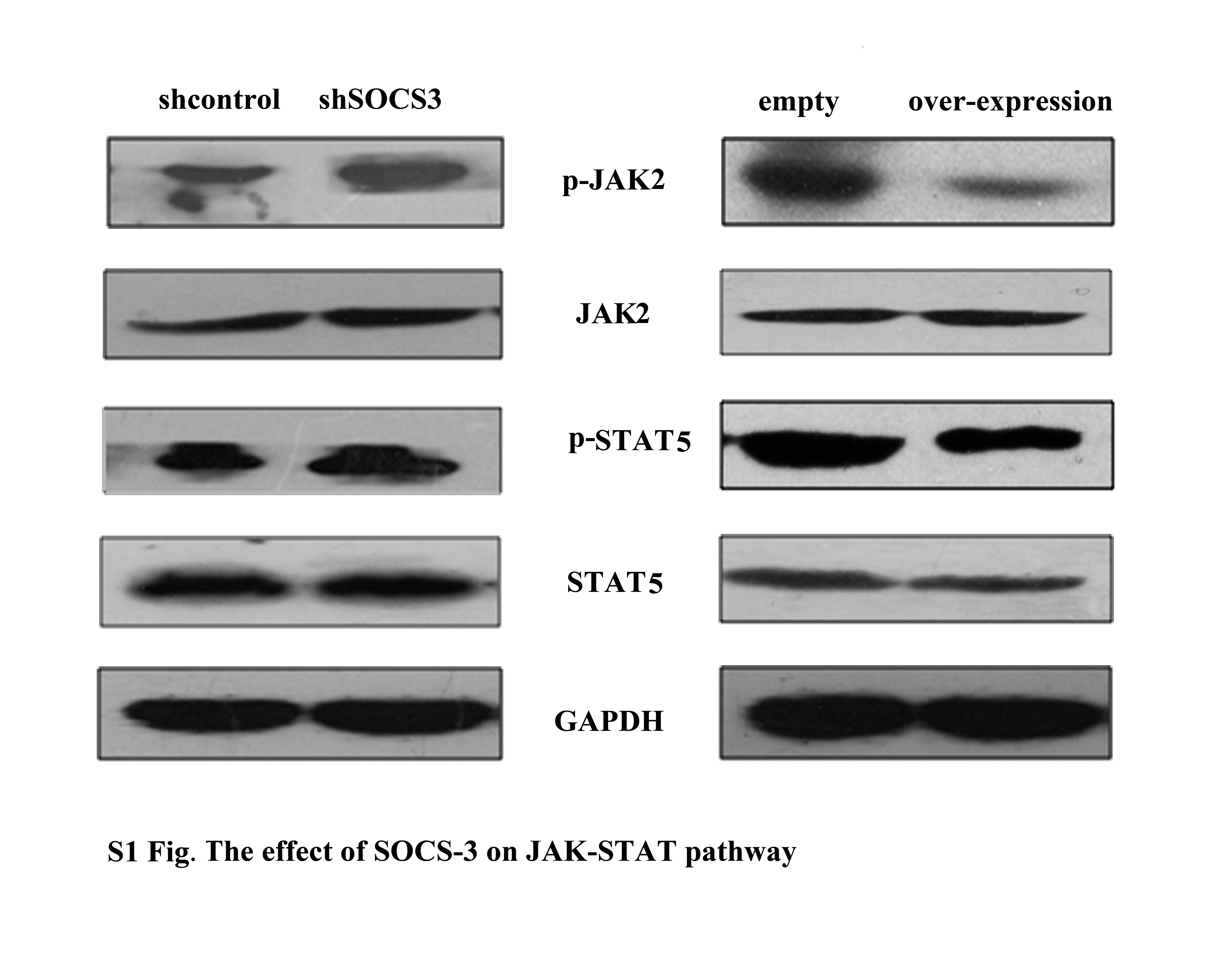

Supplement: S1 Fig — The effect of SOCS3 on JAK/STAT pathway in HSCs was examined by western-blot after SOCS3 knock-down or over-expression. (TIF) [file pone.0135259.s001.tif]

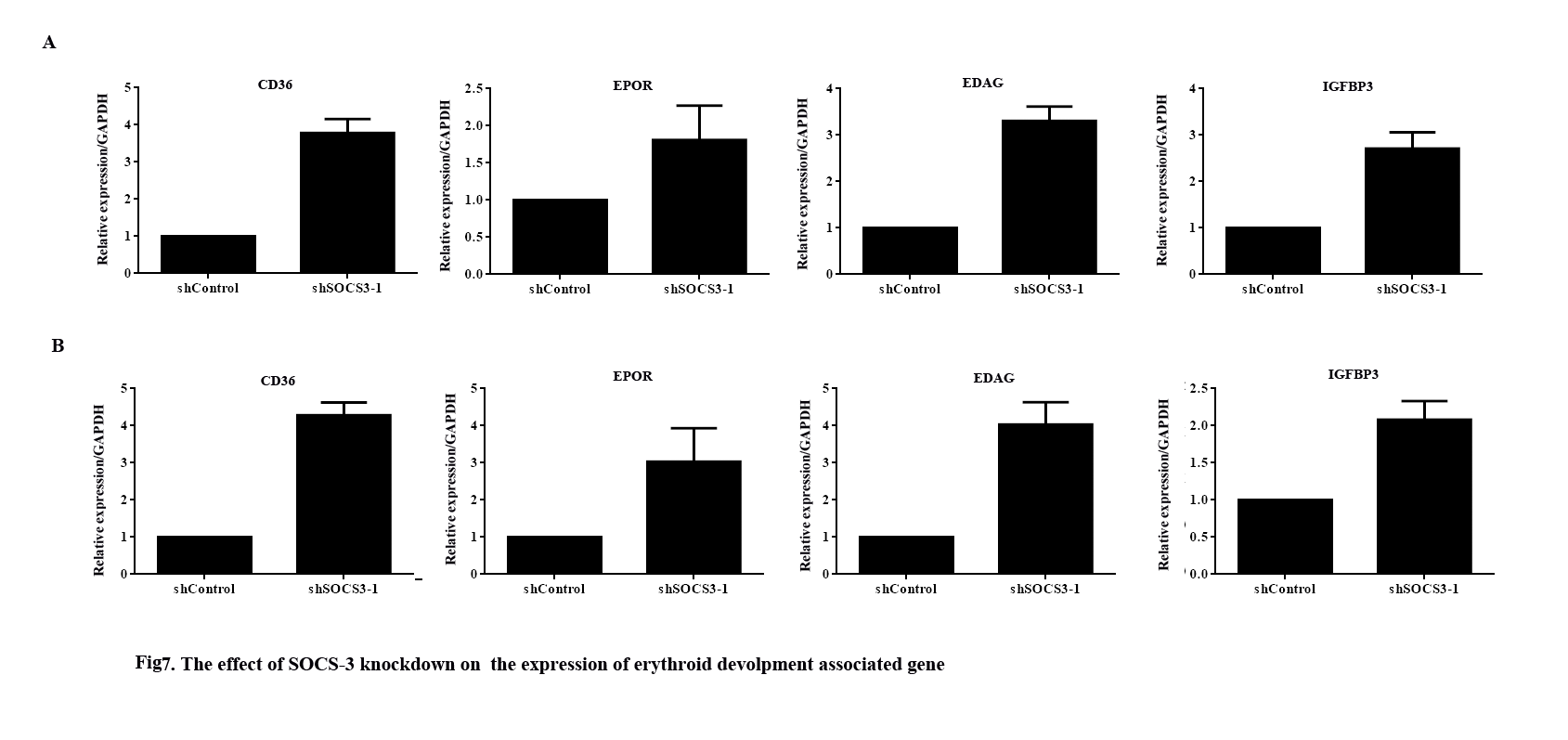

Supplement: S2 Fig — Real-time PCR analysis for the expression of CD36, HEMGN, EPOR, IGFBP in HSCs (A) or in erythroblasts on day 7 (B) in liquid culture after SOCS3 knockdown. The average of the normalized ratio of the target gene/GAPDH was calculated. The data represent the mean ± SEM from three experiments. * P<0.05 as compared with shcontrol groups. (TIF) [file pone.0135259.s002.tif]
